# Testosterone, Sex Hormone-Binding Globulin and the Metabolic Syndrome in Men: An Individual Participant Data Meta-Analysis of Observational Studies

**DOI:** 10.1371/journal.pone.0100409

**Published:** 2014-07-14

**Authors:** Judith S. Brand, Maroeska M. Rovers, Bu B. Yeap, Harald J. Schneider, Tomi-Pekka Tuomainen, Robin Haring, Giovanni Corona, Altan Onat, Marcello Maggio, Claude Bouchard, Peter C. Y. Tong, Richard Y. T. Chen, Masahiro Akishita, Jourik A. Gietema, Marie-Hélène Gannagé-Yared, Anna-Lena Undén, Aarno Hautanen, Nicolai P. Goncharov, Philip Kumanov, S. A. Paul Chubb, Osvaldo P. Almeida, Hans-Ulrich Wittchen, Jens Klotsche, Henri Wallaschofski, Henry Völzke, Jussi Kauhanen, Jukka T. Salonen, Luigi Ferrucci, Yvonne T. van der Schouw

**Affiliations:** 1 Julius Center for Health Sciences and Primary Care, University Medical Center Utrecht, Utrecht, The Netherlands; 2 Departments of EBH and operating rooms, Radboud University Nijmegen Medical Center, Nijmegen, The Netherlands; 3 School of Medicine and Pharmacology, University of Western Australia, Perth, Australia; 4 Medizinische Klinik Innenstadt der Universität München, München, Germany; 5 Institute of Public Health and Clinical Nutrition, University of Eastern Finland, Kuopio, Finland; 6 Institute of Clinical Chemistry and Laboratory Medicine, University Medicine Greifswald, Greifswald, Germany; 7 Andrology and Sexual Medicine Unit Department of Clinical Physiopathology, University of Florence, Florence, Italy; 8 Department of Cardiology, Istanbul University, Istanbul, Turkey; 9 Department of Internal Medicine and Biomedical Sciences, University of Parma, Parma, Italy; 10 Pennington Biomedical Research Center, Louisiana State University System, Baton Rouge, Louisiana, United States of America; 11 Department of Medicine and Therapeutics, Chinese University of Hong Kong, Shatin, Hong Kong, Hong Kong; 12 Department of Endocrinology, Changing General Hospital, Singapore, Singapore; 13 Department of Geriatric Medicine, Graduate School of Medicine, University of Tokyo, Tokyo, Japan; 14 Department of Medical Oncology, University Medical Center Groningen, Groningen, Netherlands; 15 Department of Endocrinology, Saint-Joseph University, Beirut, Lebanon; 16 Center for Family and Community Medicine, Stockholm, Sweden; 17 Department of Clinical Chemistry, University of Helsinki, Helsinki, Finland; 18 Endocrinology Research Center, Moscow, Russia; 19 Clinical Center of Endocrinology and Gerontology, Medical University, Sofia, Bulgaria; 20 School of Psychiatry and Clinical Neurosciences, University of Western Australia, Perth, Australia; 21 Institute of Clinical Psychology, Center of Clinical Epidemiology and Longitudinal Studies, University of Dresden, Dresden, Germany; 22 Institute for Community Medicine, University Medicine Greifswald, Greifswald, Germany; 23 MAS-Metabolic Analytical Services Oy, Helsinki, Finland; 24 Clinical Research Branch, National Institute on Aging, Baltimore, Maryland, United States of America; University of Padova, Italy

## Abstract

**Background:**

Low total testosterone (TT) and sex hormone-binding globulin (SHBG) concentrations have been associated with the metabolic syndrome (MetS) in men, but the reported strength of association varies considerably.

**Objectives:**

We aimed to investigate whether associations differ across specific subgroups (according to age and body mass index (BMI)) and individual MetS components.

**Data sources:**

Two previously published meta-analyses including an updated systematic search in PubMed and EMBASE.

**Study Eligibility Criteria:**

Cross-sectional or prospective observational studies with data on TT and/or SHBG concentrations in combination with MetS in men.

**Methods:**

We conducted an individual participant data meta-analysis of 20 observational studies. Mixed effects models were used to assess cross-sectional and prospective associations of TT, SHBG and free testosterone (FT) with MetS and its individual components. Multivariable adjusted odds ratios (ORs) and hazard ratios (HRs) were calculated and effect modification by age and BMI was studied.

**Results:**

Men with low concentrations of TT, SHBG or FT were more likely to have prevalent MetS (ORs per quartile decrease were 1.69 (95% CI 1.60-1.77), 1.73 (95% CI 1.62-1.85) and 1.46 (95% CI 1.36-1.57) for TT, SHBG and FT, respectively) and incident MetS (HRs per quartile decrease were 1.25 (95% CI 1.16-1.36), 1.44 (95% 1.30-1.60) and 1.14 (95% 1.01-1.28) for TT, SHBG and FT, respectively). Overall, the magnitude of associations was largest in non-overweight men and varied across individual components: stronger associations were observed with hypertriglyceridemia, abdominal obesity and hyperglycaemia and associations were weakest for hypertension.

**Conclusions:**

Associations of testosterone and SHBG with MetS vary according to BMI and individual MetS components. These findings provide further insights into the pathophysiological mechanisms linking low testosterone and SHBG concentrations to cardiometabolic risk.

## Introduction

The metabolic syndrome (MetS) affects approximately 25% of the adult population [1] and its prevalence is increasing worldwide [2]–[4]. MetS is associated with a twofold increase in cardiovascular disease (CVD) risk and a nearly fivefold increased risk of type 2 diabetes [5], [6]. Given its major public health impact, there is an urgent need for a better understanding of the underlying mechanisms of MetS, in particular factors driving and influencing its pathophysiology.

A large number of epidemiological studies have linked low concentrations of total testosterone (TT) and its carrier protein, sex hormone-binding globulin (SHBG), to MetS in men [7]–[15]. Despite the clear link between testosterone, SHBG and MetS, the exact nature of the observed associations remains uncertain, given the high variability in the strength of associations reported. This between-study heterogeneity can be partially explained by incomparability in study design (i.e. with regard to MetS criteria, hormone assays and sample size), but also by differences in population structure. Recent evidence suggests that associations may differ according to age and BMI, as stronger associations have been reported in young [12] and nonobese [14] men. Strength of associations may also vary across individual MetS components. Cross-sectionally, stronger associations have been reported for abdominal obesity and hypertriglyceridemia [7]-[9], [16], but conflicting data exist for other MetS components [7]–[9], [16] and no studies so far have examined these associations prospectively.

We previously re-examined the observational data on testosterone, SHBG and MetS in a literature-based meta-analysis [17], but analyses for specific subgroups and MetS components were hampered by the absence of individual data. In addition, individual studies were largely heterogeneous with regard to MetS criteria and methods used for free testosterone (FT) estimation and confounder adjustment. To conduct a more comprehensive and powerful assessment of the associations of testosterone and SHBG with MetS, we pooled the original raw data of observational studies. Such a meta-analysis of individual participant data provides a unique opportunity to 1) examine associations of testosterone and SHBG with MetS in a uniform way; 2) produce estimates for specific subgroups according to age and body mass index (BMI) and 3) determine specific MetS components through which associations with testosterone and SHBG are primarily mediated. In this article, we present the findings of this collaborative project.

## Methods

### Identification of studies

Studies were eligible for inclusion if they had data on TT and/or SHBG in combination with MetS in men using a cross-sectional or prospective design. Most studies were identified in previously published meta-analyses [17], [18]; additional studies were identified following an updated systematic search in PubMed and EMBASE (using the key words ‘metabolic syndrome’, ‘insulin resistance syndrome’ and ‘syndrome X’ combined with ‘testosterone’, ‘sex hormone-binding globulin’, ‘SHBG’, ‘androgens’, ‘sex hormones’ and ‘sex steroids’), hand searching of relevant journals and correspondence with collaborating investigators. For details on the study selection procedure (and flow diagram) we refer to our literature-based meta-analysis [17], as the same approach was used for the current analysis. Thirty-three eligible studies were identified, and communication was established with the authors of 24 studies. From these studies, four declined and 20 agreed to participate. All studies used a cross-sectional design and four studies also collected outcome data prospectively. All studies were previously published and had each received local institutional review board approvals as well as consent from participants (figure S1).

### Data collection

A study protocol was sent out to all collaborators including information on study organisation, objectives, data transfer and checking. Collaborators were asked to provide data on the following variables for each individual: waist circumference, systolic and diastolic blood pressure, high-density lipoprotein (HDL) cholesterol, triglycerides, glucose, TT and SHBG concentrations, age at recruitment, use of hormonal therapy, timing of blood sample collection and details of any overnight fast, assay methods and length of follow-up for prospective data. If available, data were also collected on ethnicity, smoking status, alcohol consumption, physical activity, BMI, insulin concentration, history of CVD, type 2 diabetes and hypertension.

The original data were checked for completeness and possible inconsistencies using the original publications. For most studies, the data provided were identical to those analysed and published previously. In the TARF (Turkish Adult Risk Factor) [19], SHIP (Study of Health in Pomerania) [12] and DETECT (Diabetes Cardiovascular Risk-Evaluation: Targets and Essential Data for Commitment of Treatment) [20], [21] cohorts additional prospective data were available that were not included in their previously published reports.

### Data processing & measures

Blood samples were mostly collected in the morning after an overnight fast. In SHIP [12] samples were collected in a non-fasting state throughout the day. In DETECT [20], [21] ∼40% of the samples were non-fasting. Not all studies performed SHBG measurements, and various assays were used for the measurement of TT and SHBG (for a full description of the assay methods and samples used for the hormone analyses, see Table S1). When both TT and SHBG were provided, FT concentrations were calculated using the equation of Vermeulen et al. [22] assuming a fixed albumin concentration of 43 g/L. We recoded categorical variables on alcohol consumption (drinker vs. non-drinker), cigarette smoking (current smoker vs. non-smoker) and physical activity (active vs. inactive) to maximize comparability across studies. When both glucose and insulin concentrations were provided, the homeostasis model assessment for insulin resistance (HOMA-IR) was calculated using the formula HOMA-IR  =  (fasting insulin in mIU/L x fasting glucose in mmol/L)/22.5. Values of HOMA-IR were not normally distributed and transformed logarithmically prior to analysis.

MetS was defined according to the most recent harmonized definition presented in the 2009 Joint Scientific Statement [23], using ethnic-specific cut-offs for abdominal obesity. Men were considered to have MetS if they had ≥3 of the following components: 1. abdominal obesity (waist circumference ≥102 cm for Caucasian men and waist circumference ≥90 cm for Asian men); 2. hypertriglyceridemia (triglycerides ≥1.7 mmol/L); 3. low HDL-cholesterol (HDL-cholesterol <1.03 mmol/L), 4. hyperglycaemia (fasting blood glucose ≥5.6 mmol/L); 5. hypertension (systolic blood pressure ≥130 mm Hg or diastolic blood pressure ≥85 mm Hg). Men taking antihypertensive medication were considered having high blood pressure and those with type 2 diabetes were counted as having hyperglycaemia. We slightly modified the criteria for men having non-fasting blood samples (using a blood glucose cut-off of ≥8.0 mmol/L and triglyceride cut-off of ≥2.3 mmol/L) [24].

### Statistical analyses

Analyses were restricted to men aged 18 years and older not using hormonal therapy (N = 14,025). We excluded men with missing data on individual MetS components (N = 1,186). We further removed extreme outliers >4 standard deviations (SD) from the mean for measured TT, SHBG, and calculated FT concentrations (N = 28), leaving 12,811 men with complete data on TT and 9,525 men with complete data on SHBG and FT, respectively. Sex hormone concentrations were categorized into quartiles using cut-off points determined separately for cross-sectional and prospective data.

We first examined the associations between sex hormones and prevalent MetS. To account for between-study heterogeneity and within study correlation, we used mixed effects logistic regression models (i.e. generalized linear mixed-effects models (GLMM) with logit link function) including a random intercept for study. In these models, odds ratios (ORs) and 95% confidence intervals (95% CIs) were estimated using the Laplace approximation [25]. Next, we studied the associations of sex hormones with incident MetS. For these analyses, we excluded all individuals with MetS at baseline. We used shared frailty models with random effects at the study level to estimate hazard ratios (HRs) and 95% CIs. The shared frailty model is an extension of the Cox proportional hazards model and accounts for within study correlation by incorporating shared random effects. We performed linear trend analysis by entering quartiles as a continuous term into the model. We also estimated ORs and HRs per quartile decrease of TT, SHBG and FT to provide a summary measure of association.

To investigate the influence of potential confounders, we calculated age-adjusted and multivariable-adjusted ORs and HRs including age and lifestyle factors (smoking status, alcohol consumption and physical activity). In a next step, we additionally adjusted the analyses for BMI and HOMA-IR to examine whether associations between sex hormones and MetS were independent of body composition and insulin resistance. To investigate whether associations of TT with MetS were influenced by SHBG, we additionally adjusted for SHBG in a separate analysis. We tested for effect modification by age and BMI by including interaction terms using the Wald-test. If a significant interaction was found, we stratified the analyses for age (<40 years, 40–60 years, >60 years) and BMI (<25 kg/m^2^, 25–30 kg/m^2^, ≥30 kg/m^2^). We also performed a series of sensitivity analyses. First, we excluded men with prevalent type 2 diabetes (diagnosed diabetes or fasting blood glucose ≥7 mmol/L) and CVD at baseline. To investigate the influence of potential selection bias, we also repeated the analyses including population-based samples only. Next, we excluded men with non-fasting blood samples to examine the impact of measurement errors due to fasting state. To assess the impact of other methodological differences between studies, we also repeated the analyses using study-specific quartiles of TT, SHBG and FT.

Finally, we examined associations with each MetS component separately. We did this analysis for both prevalent and incident MetS components. For the latter, we studied incidence of individual components after excluding men with the respective component at baseline. We used linear mixed effects models to estimate multivariable-adjusted means of TT, SHBG and FT across categories of MetS components (0, 1, 2, and ≥3).

All statistical analyses were performed using STATA version 11.1 (Stata Corp., College Station, TX, USA).

## Results

Table 1 summarizes the participant characteristics for each individual study. All men had complete data on age and history of type 2 diabetes. Nineteen studies had recorded data on BMI, 13 studies had data on insulin concentrations and CVD history and 9 studies collected data on all lifestyle factors. Absolute sex hormone concentrations varied across individual studies: variations for TT, SHBG, and FT were 1.6-fold, 2.0-fold and 2.2-fold respectively (Table S1).

**Table 1:** Participant characteristics of the included studies.

Study	Country	No. of men	Follow-up (years) Median (range)	Age (years) Mean (SD)	BMI (kg/m^2^) Mean (SD)	Insulin (mIU/L) Mean (SD)	Smoking (yes)% (n)	Alcohol drinking (yes)% (n)	Physically active (yes)% (n)	History of diabetes (yes)% (n)	History of CVD (yes)% (n)
Akishita et al, 2010 [26]	Japan	192	NA	48.8 (9.4)	25.2 (4.0)	6.7 (4.1)	44.1 (52)	NA	NA	0.0 (0)	NA
Chen et al, 2010 [27]	Singapore	206	NA	55.1 (7.1)	25.2 (3.8)	NA	22.1 (45)	29.4 (60)	51.6 (98)	15.1 (31)	NA
Haring et al, 2009 [12]	Germany	2000	5.0 (4.4–8.5)	50.8 (16.5)	27.6 (4.0)	NA	33.7 (671)	76.5 (1521)	69.9 (1390)	8.6 (172)	NA
Schneider et al, 2009 [20]	Germany	2448	4.0 (0.9–4.6)	58.7 (13.3)	27.8 (4.3)	NA	18.6 (387)	84.9 (1804)	27.1 (623)	22.9 (538)	20.3 (496)
Chubb et al, 2008 [8]	Australia	2489	NA	77.0 (3.6)	26.2 (3.5)	NA	8.4 (136)	NA	NA	0.0 (0)	37.6 (936)
Corona et al, 2008 [28]	Italy	587	NA	54.1 (12.6)	NA	NA	NA	NA	NA	27.6 (162)	15.2 (89)
Emmelot-Vonk et al, 2008 [29]	Netherlands	200	NA	67.3 (4.9)	27.3 (3.9)	9.1 (8.3)	15.0 (30)	80.5 (161)	NA	0.0 (0)	NA
Goncharov et al, 2008 [30]	Russia	60	NA	30.2 (6.4)	32.1 (2.9)	15.7 (14.5)	26.7 (16)	NA	23.3 (14)	0.0 (0)	NA
Onat et al, 2007 [19]	Turkey	564	2.0 (2.0–2.0)	53.5 (11.0)	27.7 (4.3)	10.1 (8.3)	36.9 (208)	17.4 (98)	48.1 (270)	0.0 (0)	11.7 (66)
Chen et al, 2006 [31]	Australia	60	NA	76.4 (5.2)	26.7 (3.2)	NA	NA	NA	NA	6.7 (4)	NA
Gannagé-Yared et al, 2006 [32]	Lebanon	152	NA	59.3 (7.0)	27.3 (3.7)	9.2 (4.6)	NA	NA	77.0 (117)	0.0 (0)	14.5 (22)
Maggio et al, 2006 [33]	Italy	421	NA	73.8 (6.7)	27.1 (3.3)	11.2 (6.1)	21.4 (90)	88.8 (371)	52.3 (219)	15.0 (63)	19.5 (82)
Robeva et al, 2006 [34]	Bulgaria	18	NA	31.9 (9.3)	30.0 (6.6)	14.3 (9.2)	44.4 (8)	55.6 (10)	NA	5.6 (1)	5.6 (1)
Muller et al, 2005 [10]	Netherlands	376	NA	60.0 (11.3)	26.2 (3.5)	8.6 (5.8)	24.7 (93)	84.1 (313)	66.7 (248)	5.1 (19)	16.0 (60)
Nuver et al, 2005 [35]	Netherlands	161	NA	37.9 (9.3)	25.0 (3.3)	9.3 (5.5)	36.0 (58)	NA	NA	0.6 (1)	NA
Undén et al, 2005 [36]	Sweden	137	NA	47.0 (16.2)	25.6 (3.9)	11.1 (6.2)	16.8 (23)	92.0 (126)	29.6 (40)	5.1 (7)	9.5 (13)
Tong et al, 2005 [37]	China	295	NA	41.0 (8.7)	25.3 (3.8)	NA	22.7 (67)	26.2 (77)	NA	0.0 (0)	0.0 (0)
Laaksonen et al, 2004 [15]	Finland	2028	11.2 (9.7–14.4)	52.7 (5.7)	27.0 (3.5)	11.7 (7.5)	31.4 (637)	86.6 (1753)	54.1 (1094)	5.0 (102)	36.0 (730)
Ukkola et al, 2001 [38]	Canada	321	NA	36.2 (13.7)	27.1 (5.1)	10.5 (7.2)	14.8 (47)	57.6 (183)	0.0 (0)	0.0 (0)	0.0 (0)
Hautanen et al, 2000 [39]	Finland	96	NA	45.0 (4.8)	26.3 (4.0)	9.0 (7.3)	36.5 (35)	95.0 (91)	30.2 (29)	0.0 (0)	0.0 (0)

Abbreviations: BMI  =  body mass index; CVD  =  cardiovascular disease; SD  =  standard deviation; NA  =  not available.

### Associations between sex hormones and prevalent MetS

The overall prevalence of MetS was 27.9% (N = 3,574). An inverse relation was observed between TT, SHBG, FT, and MetS (Table 2). Men with low TT concentrations were more likely to have prevalent MetS compared to men with high TT concentrations (OR per quartile decrease  = 1.70 (95% CI 1.63-1.77)). Associations were similar for SHBG (OR per quartile decrease  = 1.75 (95% CI 1.66-1.84)), but weaker for FT (OR per quartile decrease  = 1.40 (95% CI 1.32-1.47)). Adjustment for lifestyle factors did not materially change the ORs. Associations were attenuated after adjustment for BMI and HOMA-IR, but remained statistically significant (Table 2). The association between TT and MetS weakened, but persisted after adjusting for SHBG (OR per quartile decrease of TT  = 1.48 (95% CI 1.37-1.59)).

**Table 2:** Odds ratios for prevalent metabolic syndrome according to quartiles of total testosterone, SHBG and free testosterone – results from cross-sectional studies.

	OR (95% CI)						
	Total dataset	Subset data (lifestyle factors)		Subset data (BMI)		Subset data (HOMA-IR)	
	Model 1	Model 1	Model 2	Model 1	Model 3	Model 1	Model 4
Total testosterone	N = 12811	N = 8094	N = 8094	N = 8066	N = 8066	N = 3724	N = 3724
Q1 (<12.4 nmol/L)	5.07 (4.45-5.80)	4.84 (4.13–5.68)	4.93 (4.20–5.79)	4.84 (2.13–5.68)	2.79 (2.34–3.33)	4.69 (3.65–6.02)	1.93 (1.44–2.59)
Q2 (12.4–15.9 nmol/L)	2.84 (2.49–3.25)	2.70 (2.30–3.18)	2.75 (2.33–3.24)	2.72 (2.31–3.20)	1.83 (1.53–2.19)	2.92 (2.27–3.76)	1.56 (1.17–2.09)
Q3 (16.0–20.4 nmol/L)	1.83 (1.59–2.10)	1.75 (1.49–2.06)	1.77 (1.50–2.09)	1.75 (1.49–2.07)	1.37 (1.14–1.64)	1.70 (1.33–2.19)	1.20 (0.90–1.59)
Q4 (>20.4 nmol/L)	1.00 (REF)	1.00 (REF)	1.00 (REF)	1.00 (REF)	1.00 (REF)	1.00 (REF)	1.00 (REF)
*P* (trend)	<0.001	<0.001	<0.001	<0.001	<0.001	<0.001	<0.001
							
per quartile decrease	1.70 (1.63–1.77)	1.68 (1.60–1.76)	1.69 (1.60–1.77)	1.68 (1.60–1.76)	1.41 (1.33–1.49)	1.68 (1.55–1.81)	1.25 (1.14–1.37)
SHBG	N = 9525	N = 5552	N = 5552	N = 5547	N = 5547	N = 3304	N = 3304
Q1 (<28.8 nmol/L)	5.26 (4.46–6.20)	5.03 (4.08–6.21)	5.18 (4.19–6.40)	5.04 (4.09–6.22)	2.96 (2.34–3.75)	5.77 (4.27–7.79)	2.61 (1.84–3.71)
Q2 (28.8–38.6 nmol/L)	2.93 (2.50–3.45)	3.08 (2.51–3.79)	3.13 (2.54–3.84)	3.08 (2.51–3.79)	2.31 (1.84–2.90)	3.61 (2.66–4.88)	2.35 (1.66–3.33)
Q3 (38.7–51.2 nmol/L)	1.67 (1.42–1.97)	1.74 (1.42–2.13)	1.75 (1.43–2.15)	1.73 (1.41–2.12)	1.44 (1.15–1.80)	1.60 (1.16–2.20)	1.21 (0.84–1.74)
Q4 (>51.2 nmol/L)	1.00 (REF)	1.00 (REF)	1.00 (REF)	1.00 (REF)	1.00 (REF)	1.00 (REF)	1.00 (REF)
*P* (trend)	<0.001	<0.001	<0.001	<0.001	<0.001	<0.001	<0.001
							
per quartile decrease	1.75 (1.66–1.84)	1.72 (1.61–1.83)	1.73 (1.62–1.85)	1.72 (1.61–1.84)	1.45 (1.34–1.56)	1.83 (1.67–2.01)	1.41 (1.27–1.58)
Free testosterone	N = 9525	N = 5552	N = 5552	N = 5547	N = 5547	N = 3304	M = 3304
Q1 (<236 pmol/L)	2.75 (2.32–3.27)	3.16 (2.53–3.96)	3.13 (2.50–3.92)	3.16 (2.53–3.95)	1.99 (1.55–2.56)	2.88 (2.16–3.84)	1.23 (0.87–1.75)
Q2 (236–303 pmol/L)	2.08 (1.76–2.45)	2.37 (1.91–2.93)	2.35 (1.89–2.91)	2.36 (1.91–2.93)	1.68 (1.32–2.13)	2.31 (1.78–3.00)	1.26 (0.92–1.72)
Q3 (304–383 pmol/L)	1.47 (1.25–1.73)	1.53 (1.25–1.89)	1.53 (1.24–1.88)	1.54 (1.25–1.89)	1.27 (1.01–1.60)	1.57 (1.24–1.99)	1.09 (0.83–1.43)
Q4 (>383 pmol/L)	1.00 (REF)	1.00 (REF)	1.00 (REF)	1.00 (REF)	1.00 (REF)	1.00 (REF)	1.00 (REF)
*P* (trend)	<0.001	<0.001	<0.001	<0.001	<0.001	<0.001	0.15
							
per quartile decrease	1.40 (1.32–1.47)	1.47 (1.37–1.58)	1.46 (1.36–1.57)	1.47 (1.37–1.58)	1.26 (1.16–1.37)	1.44 (1.31–1.57)	1.08 (0.97–1.21)

Abbreviations: BMI  =  body mass index; HOMA-IR  =  homeostasis model assessment for insulin resistance; SHBG  =  sex hormone-binding globulin; OR  =  odds ratio; CI  =  confidence interval. Lifestyle factors: smoking, alcohol consumption and physical activity.

Model 1: adjusted for age.

Model 2: Model 1 plus lifestyle factors.

Model 3: Model 2 plus BMI.

Model 4: Model 3 HOMA-IR.

Results from models including interaction terms are shown in Table 3. The association between SHBG and MetS was modified by BMI. The association with SHBG was stronger in men with a lower BMI (*P* for interaction  = 0.03). Associations with TT and FT were not modified by BMI. We also observed a significant interaction with age. Associations of TT and FT with MetS were stronger in men aged <40 years (*P* for interaction  = 0.004 and 0.01 respectively).

**Table 3:** Odds ratios for prevalent metabolic syndrome per quartile decrease of total testosterone, SHBG and free testosterone, stratified by age and BMI – results from cross-sectional studies.

	OR (95% CI)		
	Total testosterone	SHBG	Free testosterone
Body mass index			
<25 kg/m^2^	N = 2377	N = 1688	N = 1688
	1.43 (1.24–1.65)	2.20 (1.77–2.72)	1.22 (0.97–1.53)
25–30 kg/m^2^	N = 3969	N = 2714	N = 2714
	1.51 (1.40–1.62)	1.50 (1.35–1.66)	1.29 (1.16–1.44)
>30 kg/m^2^	N = 1720	N = 1145	N = 1145
	1.37 (1.24–1.51)	1.33 (1.18–1.50)	1.31 (1.15–1.50)
*P* interaction	0.40	0.003	0.67
Age			
<40 years	N = 1080	N = 875	N = 875
	1.87 (1.57–2.22)	1.50 (1.22–1.84)	1.57 (1.29–1.91)
40–60 years	N = 3985	N = 3185	N = 3185
	1.78 (1.65–1.92)	1.68 (1.54–1.83)	1.52 (1.38–1.66)
≥60 years	N = 3029	N = 1492	N = 1492
	1.54 (1.43–1.66)	1.61 (1.43–1.82)	1.32 (1.16–1.50)
*P* interaction	0.004	0.11	0.01

Odds ratios are adjusted for age, smoking, alcohol consumption and physical activity. Abbreviations: SHBG  =  sex hormone-binding globulin; OR  =  odds ratio; CI  =  confidence interval.

### Associations between sex hormones and incident MetS

In total, 584 incident MetS cases were documented during 17,625 person years of follow-up. Men with low sex hormone concentrations at baseline had an increased risk of incident MetS at follow-up (Table 4). HRs per quartile decrease were 1.24 (95% CI 1.16-1.35), 1.43, (95% CI 1.29-1.59) and 1.14 (95% CI 1.01-1.29) for TT, SHBG and FT respectively. Again, adjustment for lifestyle factors had little effect, but associations weakened after further adjustment for BMI and HOMA-IR. In particular, associations for FT were no longer significant after adjustment for BMI (Table 4). The association with TT was attenuated, but remained significant after adjustment for SHBG (HR per quartile decrease of TT  = 1.13 (95% CI 1.01-1.27)).

**Table 4:** Hazard ratios for incident metabolic syndrome according to quartiles of total testosterone, SHBG and free testosterone – results from prospective studies.

	HR (95% CI)						
	Total dataset	Subset data (lifestyle factors)		Subset data (BMI)		Subset data (HOMA-IR)	
	Model 1	Model 1	Model 2	Model 1	Model 3	Model 1	Model 4
Total testosterone	N = 3022	N = 2941	N = 2941	N = 2933	N = 2933	N = 792	N = 792
Q1 (<13.4 nmol/L)	2.01 (1.56–2.59)	2.00 (1.55–2.59)	2.02 (1.56–2.62)	2.01 (1.55–2.59)	1.48 (1.13–1.92)	2.56 (1.60–4.11)	1.66 (1.01–2.72)
Q2 (13.4–17.0 nmol/L)	1.84 (1.42–2.38)	1.81 (1.40–2.35)	1.83 (1.41–2.38)	1.80 (1.39–2.33)	1.52 (1.17–1.99)	2.68 (1.71–4.19)	1.75 (1.10–2.80)
Q3 (17.1–21.4 nmol/L)	1.42 (1.09–1.86)	1.40 (1.07–1.83)	1.40 (1.07–1.83)	1.39 (1.07–1.82)	1.23 (0.94–1.61)	1.73 (1.09–2.75)	1.24 (0.77–1.99)
Q4 (>21.4 nmol/L)	1.00 (REF)	1.00 (REF)	1.00 (REF)	1.00 (REF)	1.00 (REF)	1.00 (REF)	1.00 (REF)
*P* (trend)	<0.001	<0.001	<0.001	<0.001	0.002	<0.001	0.02
							
per quartile decrease	1.24 (1.16–1.35)	1.25 (1.16–1.35)	1.25 (1.16–1.36)	1.25 (1.16–1.35)	1.14 (1.05–1.23)	1.39 (1.20–1.59)	1.20 (1.03–1.40)
SHBG	N = 1899	N = 1894	N = 1894	N = 1892	N = 1892	N = 788	N = 788
Q1 (<30.7 nmol/L)	2.98 (2.15–4.12)	2.96 (2.14–2.09)	3.02 (2.18–4.19)	2.96 (2.14–4.09)	2.06 (1.46–2.89)	3.45 (1.82–6.53)	1.95 (1.00–3.81)
Q2 (30.7–41.1 nmol/L)	1.89 (1.37–2.61)	1.88 (1.36–2.59)	1.90 (1.38–2.63)	1.88 (1.36–2.59)	1.51 (1.09–2.10)	2.03 (1.05–3.92)	1.31 (0.67–2.55)
Q3 (41.2–56.4 nmol/L)	1.45 (1.05–1.98)	1.45 (1.05–1.98)	1.45 (1.05–1.98)	1.44 (1.05–1.98)	1.22 (0.89–1.68)	1.44 (0.69–2.98)	1.08 (0.52–2.24)
Q4 (>56.4 nmol/L)	1.00 (REF)	1.00 (REF)	1.00 (REF)	1.00 (REF)	1.00 (REF)	1.00 (REF)	1.00 (REF)
*P* (trend)	<0.001	<0.001	<0.001	<0.001	<0.001	<0.001	0.01
							
per quartile decrease	1.43 (1.29–1.59)	1.43 (1.29–1.58)	1.44 (1.30–1.60)	1.43 (1.29–1.59)	1.27 (1.14–1.42)	1.59 (1.29–1.84)	1.30 (1.07–1.58)
Free testosterone	N = 1899	N = 1894	N = 1894	N = 1892	N = 1892	N = 788	N = 788
Q1 (<238 pmol/L)	1.46 (1.00–2.12)	1.45 (0.99–2.11)	1.44 (0.99–2.10)	1.45 (0.99–2.11)	1.27 (0.87–1.85)	2.06 (0.97–4.36)	1.66 (0.76–3.63)
Q2 (238–313 pmol/L)	1.54 (1.11–2.13)	1.53 (1.11–2.12)	1.53 (1.10–2.11)	1.53 (1.11–2.12)	1.43 (1.03–1.98)	1.64 (1.05–2.56)	1.56 (0.99–2.48)
Q3 (314–397 pmol/L)	1.24 (0.91–1.68)	1.24 (0.91–1.69)	1.24 (0.91–1.69)	1.24 (0.92–1.69)	1.28 (0.95–1.74)	1.41 (0.98–2.05)	1.22 (0.84–1.78)
Q4 (>397 pmol/L)	1.00 (REF)	1.00 (REF)	1.00 (REF)	1.00 (REF)	1.00 (REF)	1.00 (REF)	1.00 (REF)
*P* (trend)	0.03	0.03	0.04	0.03	0.19	0.01	0.04
							
per quartile decrease	1.14 (1.01–1.29)	1.14 (1.01–1.28)	1.14 (1.01–1.28)	1.14 (1.01–1.28)	1.08 (0.96–1.22)	1.28 (1.06–1.54)	1.22 (1.01–1.48)

Abbreviations: BMI  =  body mass index; HOMA-IR  =  homeostasis model assessment for insulin resistance; SHBG  =  sex hormone-binding globulin; HR  =  hazard ratio; CI  =  confidence interval. Lifestyle factors: smoking, alcohol consumption and physical activity.

Model 1: adjusted for age.

Model 2: Model 1 plus lifestyle factors.

Model 3: Model 2 plus BMI.

Model 4: Model 3 HOMA-IR.

Interaction analyses showed that the association between TT and MetS was strongest in men with a BMI <25 kg/m^2^ (Table 5, *P* for interaction  = 0.02). Although no signification interaction between SHBG and BMI was observed, there was some evidence of a U-shaped relation with associations being strongest in men <25 kg/m^2^ (Table 5). In contrast to the cross-sectional data, no effect modification by age was observed in prospective analyses.

**Table 5:** Hazard ratios for incident metabolic syndrome per quartile decrease of total testosterone, SHBG and free testosterone, stratified by age and BMI – results from prospective studies.

	HR (95% CI)		
	Total testosterone	SHBG	Free testosterone
Body mass index			
<25 kg/m^2^	N = 1045	N = 625	N = 625
	1.58 (1.25–2.00)	1.59 (1.15–2.21)	1.16 (0.79–1.69)
25–30 kg/m^2^	N = 1546	N = 1028	N = 1028
	1.08 (0.98–1.19)	1.17 (1.03–1.33)	1.09 (0.94–1.26)
≥30 kg/m^2^	N = 342	N = 239	N = 239
	1.13 (0.95–1.36)	1.49 (1.16–1.93)	1.12 (0.85–1.48)
*P* interaction	0.02	0.65	0.62
Age			
<40 years	N = 487	N = 372	N = 372
	1.24 (1.00–1.53)	1.39 (1.09–1.77)	100 (0.77–1.29)
40–60 years	N = 1449	N = 1027	N = 1027
	1.27 (1.14–1.42)	1.32 (1.15–1.51)	1.22 (1.04–1.43)
≥60 years	N = 1005	N = 495	N = 495
	1.19 (1.04–1.35)	1.32 (1.07–1.62)	1.11 (0.87–1.43)
*P* interaction	0.31	0.53	0.45

Hazard ratios are adjusted for age, smoking, alcohol consumption and physical activity. Abbreviations: SHBG  =  sex hormone-binding globulin; OR  =  odds ratio; CI  =  confidence interval.

We repeated all analyses first, using non-fasting blood samples and second, after excluding men with a history of type 2 diabetes and CVD. Estimates were not materially different in these sensitivity analyses. Results remained also unchanged in analyses using study-specific quartiles and analyses restricted to population-based samples (data not shown).

### Associations between sex hormones and number of MetS components

**Figure 1:**
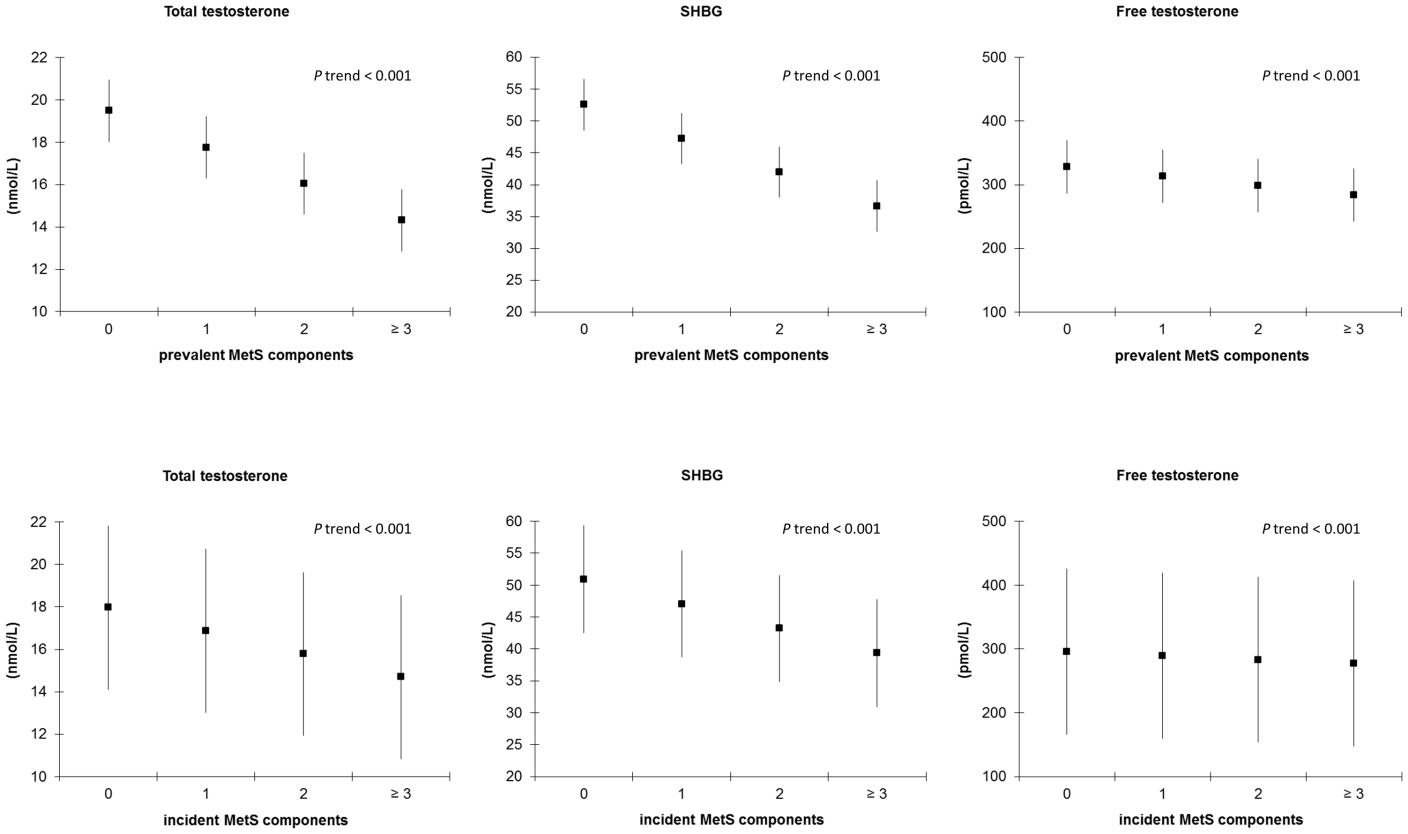
Sex hormone concentrations by number of prevalent and incident metabolic syndrome components – results from cross-sectional and prospective studies. Multivariable adjusted means and 95% confidence intervals for sex hormone concentrations by number of prevalent and incident metabolic syndrome components and the *P* value for linear trend. Abbreviations: SHBG  =  sex hormone-binding globulin. Means are adjusted for age, smoking, alcohol consumption and physical activity.

Figure 1 shows the mean concentrations of TT, SHBG, and FT according to the number of MetS components. In cross-sectional analyses, TT, SHBG, and FT concentrations decreased gradually with increasing number of prevalent MetS components (*P* trend <0.001). Although differences in sex hormone concentrations were smaller for incident MetS components, a gradual linear decrease of TT, SHBG, and FT was observed as the number of components increased (Figure 1).

### Associations between sex hormones and individual MetS components

**Figure 2:**
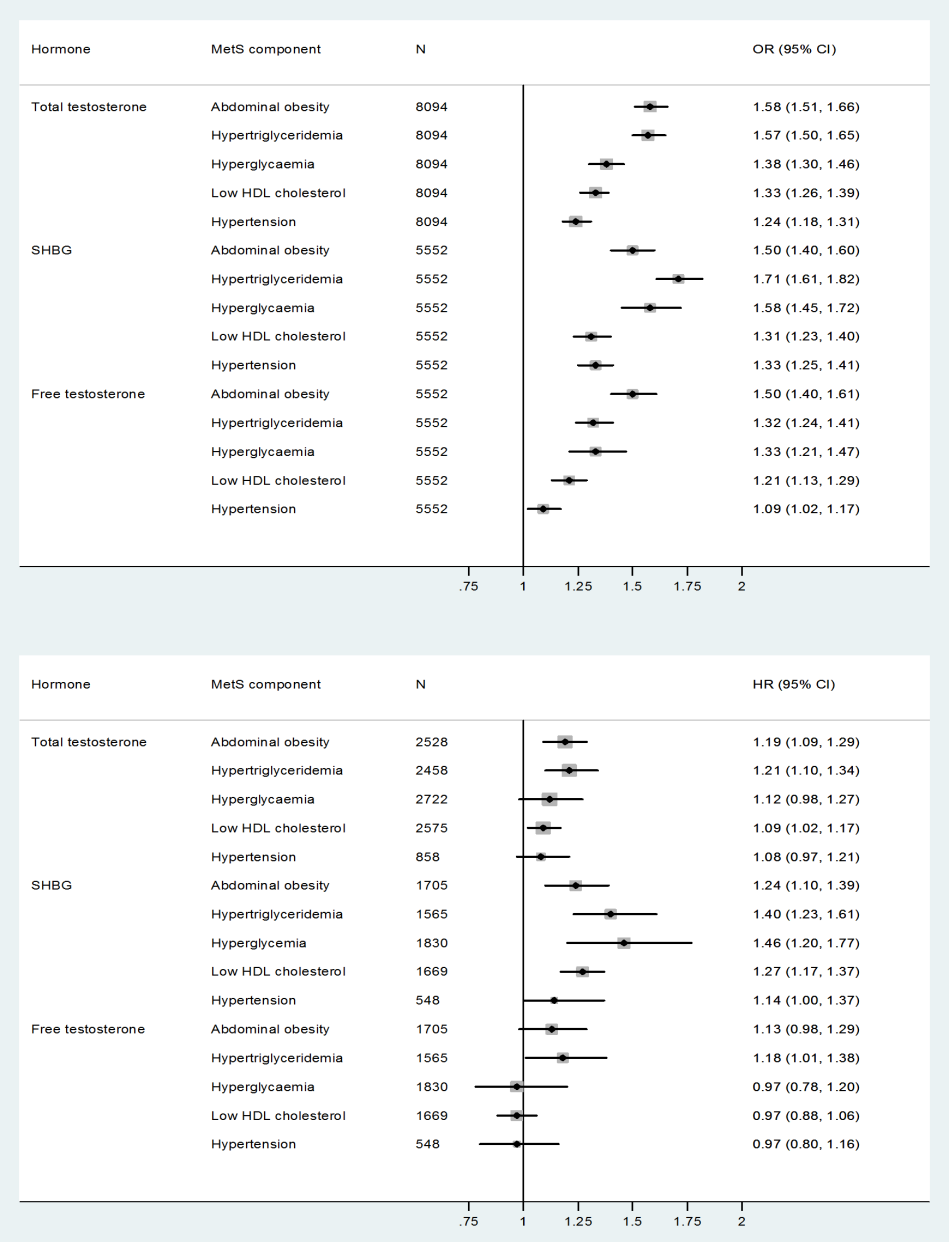
Odds ratios and hazard ratios for individual metabolic syndrome components per quartile decrease of total testosterone, SHBG and free testosterone. Models are adjusted for age, smoking, alcohol consumption and physical activity. Abbreviations: SHBG  =  sex hormone-binding globulin; OR  =  odds ratio; HR  =  hazard ratio; CI  =  confidence interval.

Figure 2 shows the multivariable-adjusted ORs for each prevalent MetS component. Associations with TT were strongest for prevalent abdominal obesity (OR per quartile decrease  = 1.58 (95% CI 1.51-1.66)) and hypertriglyceridemia (OR per quartile decrease  = 1.57 (95% CI 1.50-1.65)), and weakest for prevalent hypertension (OR per quartile decrease  = 1.24 (95% CI 1.18-1.31)). A similar pattern was observed for SHBG and FT, with the exception that low FT and SHBG concentrations were also strongly linked to prevalent hyperglycaemia (Figure 2).

Differences in strength were less marked for incident MetS components, although a similar pattern for TT was observed. Low TT concentrations at baseline were most strongly associated with incident abdominal obesity (HR per quartile decrease  = 1.19 (95% CI 1.09-1.29)) and hypertriglyceridemia (HR per quartile decrease  = 1.21 (95% CI 1.10-1.34)). Low baseline SHBG concentrations were associated with all incident MetS components. Associations were strongest for incident hyperglycaemia (HR per quartile decrease  = 1.46 (95% CI 1.20-1.77)) and hypertriglyceridemia (HR per quartile decrease  = 1.40 (95% CI 1.23-1.61)). Low FT concentrations were associated with incident hypertriglyceridemia (HR  = 1.18 (95% CI 1.01-1.38)) and abdominal obesity (HR  = 1.13 (95% CI 0.98-1.29)), although the latter was not statistically significant.

## Discussion

In this unique meta-analysis of individual participant data, we found that men with low concentrations of TT, SHBG and FT were more likely to have MetS compared to those having high sex hormone concentrations. The revealed associations were independent of age and lifestyle factors and were weaker for incident than prevalent MetS. SHBG was the main determinant of incident MetS, but adjustment for SHBG did not fully explain associations of TT with MetS. Associations of testosterone and SHBG with MetS were strongest in non-overweight men and abdominal obesity, hypertriglyceridemia and hyperglycaemia were the main drivers of the overall associations found.

The major strength of our study was that by re-analysing the individual data from 20 observational studies, we were able to study relevant subgroups and individual MetS components with sufficient statistical power. Furthermore, the use of raw data enabled us to apply consistent methods for MetS assessment and FT estimation, and to adjust for potential confounders in a uniform way. Nevertheless, some potential limitations should be discussed. First, not all eligible studies participated in this collaborative meta-analysis, which may have introduced ‘collaboration bias’, a term equivalent to publication bias in literature-based meta-analyses. However, we think that reasons to participate are pragmatic, not related to either determinant or outcome status, therefore minimizing the likelihood of this bias. Second, individual studies were methodologically heterogeneous; confounder and outcome data were not collected in a standardised way. Our statistical approach accounted for these methodological differences between studies by incorporating random effects at the study level. Third, all studies used commercially available immunoassays for the measurement of testosterone and SHBG. These assays lack reliability in the lower end of the distribution [40], but over a wide range of concentrations their measures correlate well with those obtained with mass spectrometry [41]–[45]. Also, the diversity of immunoassays used will not have a major impact on our risk estimates, since different assays are likely to classify subjects in the same quartile. Previous studies have shown that associations with known metabolic determinants do not heavily depend on the assay being used [45], [46]. Moreover, measurement errors resulting from interlaboratory assay differences are likely to be random, and may have resulted in underestimated associations rather than producing spurious ones [46]. Another limitation is that FT concentrations were not measured in our study but calculated using the algorithm of Vermeulen [22]. This algorithm gives a reasonable approximation of serum FT concentrations in men [22], but the level of agreement depends on the testosterone and SHBG assay being used [47]. Therefore, random measurements errors in FT are expected to be larger due to interlaboratory differences in both TT and SHBG assays. However, when we repeated the analysis using study-specific hormone quartiles, results did not change substantially, indicating that assay heterogeneity does not have a major impact on our findings. Nonetheless, more efforts are needed to increase the accuracy and standardization of sex hormone measurements. This is particularly relevant when using hormone measurements for individual diagnoses and treatment decisions, which require methods with high accuracy and precision at the lower end of the distribution. Fourth, twenty-four percent of all participants had non-fasting blood samples. In our analysis, we adjusted for fasting state by using sample-specific cut-offs. Since results were not materially different in analyses excluding non-fasting samples, we consider differential misclassification due to fasting state negligible. Finally, sex hormones were measured only once at baseline in each individual study, precluding us from studying time-related changes in sex hormone concentrations and MetS risk.

Notwithstanding the prospective design, we cannot draw definitive conclusions on the causal directionality of the observed associations. Stronger associations of sex hormones with prevalent than incident MetS suggest that low testosterone and SHBG are merely a result rather than cause of MetS. Indeed, weight loss and maintenance have been associated with an increase in testosterone and SHBG concentrations in obese men with MetS [48], [49]. Likewise, experimental studies show suppressive effects of adiposity and insulin resistance on testosterone production in men [50], [51]. On the other hand, testosterone and SHBG may also influence MetS etiology. Polymorphisms in the SHBG gene have been associated with risk of type 2 diabetes, suggesting a causal role for SHBG in metabolic disease risk [52], [53]. Moreover, a recent meta-analysis of the few available testosterone supplementation studies shows that testosterone therapy is associated with a significant reduction of fasting glucose, HOMA-IR, triglycerides and waist circumference as well as an increase of HDL-cholesterol [18]. Thus, observational and experimental data point to bidirectional relationships between sex hormones and MetS.

Adjustment for lifestyle factors had little effect on the observed associations of TT, SHBG and FT with MetS, but the strength of associations was nearly halved after adjustment for BMI and HOMA-IR. The major impact of body composition and insulin resistance was expected, as both factors represent the core abnormality of MetS [54]. Hence, adjusting for BMI and HOMA-IR may represent overadjustment. Consistent with our literature-based meta-analysis [17], we found an increase in MetS incidence with lower FT concentrations. Associations with TT and MetS remained also significant after adjusting for SHBG. These findings are important because they show that the association between testosterone and MetS cannot solely be attributed to SHBG. The fact that previous studies have reported conflicting results for FT [7]–[9], [14], [15], [55], might be due to differences in sample size and handling of potential confounders as described above. The large sample size of the present pooled meta-analysis enhanced the statistical power to detect small to moderate associations between FT and MetS.

Apart from being associated with MetS as an entity, sex hormones also show an inverse association with the number of MetS components. Previous data regarding this association are limited. In the BACH study [9], the largest difference in sex hormones was found between men having one vs. two MetS components, suggesting a decline in sex hormone concentrations before the actual onset of MetS. Our results do not support such a threshold effect, as all sex hormones decreased gradually with increasing number of MetS components. Among the five components, TT was most strongly associated with hypertriglyceridemia and abdominal obesity. A similar pattern was found previously in cross-sectional studies [7]–[9], [16], but this is the first study showing such a relationship with incident MetS components. Apart from hypertriglyceridemia and abdominal obesity, SHBG and FT were also strongly associated with hyperglycaemia.

Interestingly, we found that the association between TT and incident MetS was strongest in men with a BMI <25 kg/m^2^. The reason for this interaction is not clear, but the weaker association in overweight men suggests a dominant role for non-androgenic risk factors in this specific subgroup. This finding may also indicate the emergence of relative androgen insensitivity with increasing BMI. In children an inverse association between BMI and androgen receptor sensitivity has been reported [56], but no studies so far have explored this association in middle-aged and older men. Cross-sectionally, we found that SHBG was more strongly associated with prevalent MetS in men with a lower BMI. However, a clear interaction with BMI could not be confirmed for incident MetS. Previously, a subgroup effect of BMI has been demonstrated in relation to leptin [57], with associations of SHBG and FT being absent in obese men. Leptin resistance becomes more prevalent with increasing BMI [58], providing an explanation for the weaker associations found in overweight men. Another explanation for the observed interactions with BMI is the higher imprecision of hormone assays toward the lower end of the hormone distribution. Testosterone and SHBG concentrations decrease with increasing BMI and associations may thus be more difficult to detect in subgroups of overweight and obese men. We also found an interaction between testosterone and age when analyzing prevalent MetS, but this interaction could not be confirmed for incident MetS.

In conclusion, we observed a robust, dose-response relationship of low testosterone and SHBG concentrations with prevalent and incident MetS in men, with associations being primarily mediated through abdominal obesity, hypertriglyceridemia and hyperglycaemia. The weaker associations observed in overweight men warrant further investigation as this specific subgroup may represent a target for future prevention and intervention. Altogether, these findings provide more insight into the biological mechanisms linking low testosterone and SHBG to MetS.

### Data Availability Statement

For all individual studies, contact and agreement with local Steering Committees is required for access to individual participant data. Data can only be provided to third parties after approval from local Steering Committees, due to confidentiality agreements with study participants.

## Supporting Information

Figure S1 **Prisma Flow Diagram.**
(DOCX)

Table S1 **Assays and samples used for hormone analyses per study.**
(DOC)

Checklist S1 **PRISMA Checklist.**
(DOCX)
