# Supplementary material for: Testosterone, Sex Hormone-Binding Globulin and the Metabolic Syndrome in Men: An Individual Participant Data Meta-Analysis of Observational Studies
Source: PLoS One. 2014 Jul 14;9(7):e100409. doi: 10.1371/journal.pone.0100409 (PMC4096400; doi:10.1371/journal.pone.0100409)
Supplement: Figure S1 — Prisma Flow Diagram. (DOCX) [file pone.0100409.s001.docx]

**Figure S1 PRISMA 2009 Flow Diagram**

Studies included in quantitative synthesis (meta-analysis)
(n = 20 )

Studies included in qualitative synthesis
(n = 38)

Full-text articles excluded, with reasons
(n = 130)

Full-text articles assessed for eligibility
(n = 168 )

Records excluded
(n = 428)

Records screened
(n = 596 )

Records after duplicates removed
(n = 596 )

Additional records identified through other sources
(n = 9)

## Identification

## Eligibility

## Included

## Screening

Records identified through database searching
(n = 1042 )
