# Supplementary material for: Testosterone, Sex Hormone-Binding Globulin and the Metabolic Syndrome in Men: An Individual Participant Data Meta-Analysis of Observational Studies
Source: PLoS One. 2014 Jul 14;9(7):e100409. doi: 10.1371/journal.pone.0100409 (PMC4096400; doi:10.1371/journal.pone.0100409)
Supplement: Table S1 — Assays and samples used for hormone analyses per study. (DOC) [file pone.0100409.s002.doc]

**Supplementary Table 1.** Assays and samples used for hormone analyses per study.

| **Study** | **Testosterone assay** | **SHBG assay** | **Fasting, % (n)** | **Timing blood sample collection** | **Total testosterone (nmol/L)**  **Mean (SD)** | **SHBG**  **(nmol/L)**  **Mean (SD)** | **Free testosterone**  **(pmol/L)**  **Mean (SD)** |
| --- | --- | --- | --- | --- | --- | --- | --- |
| Akishita et al, 2010 [26] | RIA commercial kit  - radioimmunoassay | NA | 100 (192) | 7.00 a.m. - 9.00 a.m. | 19.1 (6.2) | NA | NA |
| Chen et al, 2010 [27] | Roche Elecsys  - electrochemiluminescent immunoassay | Roche Elecsys  - electrochemiluminescent immunoassay | 100 (206) | 9.00 a.m. | 15.8 (5.3) | 37.0 (15.3) | 303.2 (77.5) |
| Haring et al, 2009 [12] | Immulite  - chemiluminescent immunoassay | NA | 0 (0) | 7.00 a.m. - 4.00 p.m. | 16.6 (5.8) | 49.7 (20.6) | 273.1 (99.6) |
| Schneider et al, 2009 [20] | Roche Elecsys  - electrochemiluminescent immunoassay | NA | 58.5 (1433) | 4.00 a.m. – 21.00 p.m. | 15.3 (6.5) | NA | NA |
| Chubb et al, 2008 [8] | Immulite  - chemiluminescent immunoassay | Immulite  - chemiluminescent immunoassay | 100 (2490) | 8.00 a.m. – 10.30 a.m. | 16.0 (5.4) | 43.0 (15.6) | 283.7 (84.7) |
| Corona et al, 2008 [28] | Roche Elecsys  - electrochemiluminescent immunoassay | Roche Elecsys  - electrochemiluminescent immunoassay | 100 (558) | NA | 15.8 (6.6) | 35.4 (16.6) | 321.0 (132.9) |
| Emmelot-Vonk et al, 2008 [29] | Immulite  - chemiluminescent immunoassay | Immulite  - chemiluminescent immunoassay | 100 (200) | 8.00 a.m. - 11.00 a.m. | 13.2 (2.4) | 33.1 (10.3) | 272.4 (59.4) |
| Goncharov et al, 2008 [30] | Vitros Eci  - electrochemiluminescent immunoassay | AutoDelfia  - fluoroimmunoassay | 100 (60) | 8.30 a.m. – 10.30 a.m. | 13.4 (5.9) | 34.4 (22.8) | 276.6 (112.0) |
| Onat et al, 2007 [19] | Roche Elecsys  - electrochemiluminescent immunoassay | Roche Elecsys  - electrochemiluminescent immunoassay | 95.7 (536) | 8.00 a.m. – 10.00 a.m. | 12.9 (7.7) | 44.6 (20.4) | 226.4 (137.4) |
| Chen et al, 2006 [31] | Roche Elecsys  - electrochemiluminescent immunoassay | NA | 100 (60) | NA | 13.1 (4.6) | NA | NA |
| Gannagé-Yared et al, 2006 [32] | Immulite  - chemiluminescent immunoassay | Immulite  - chemiluminescent immunoassay | 100 (152) | 8.00 a.m. – 9.00 a.m. | 13.3 (4.1) | 36.7 (14.8) | 253.7 (69.9) |
| Maggio et al, 2006 [33] | Diagnostic Systems Laboratories  - radioimmunoassay | NA | 100 (421) | 7.00 a.m. - 8.00 a.m. | 15.0 (4.5) | NA | NA |
| Robeva et al, 2006 [34] | RIA commercial kit  - radioimmunoassay | NA | 100 (18) | 8.00 a.m. – 9.00 a.m. | 16.9 (7.7) | NA | NA |
| Muller et al, 2005 [10] | In-house competitive RIA  - radioimmunoassay | Immulite  - immunoradiometric assay | 99.5 (374) | 8.00 a.m. - 10.00 a.m. | 18.6 (5.4) | 40.6 (14.4) | 352.1 (98.5) |
| Nuver et al, 2005 [35] | RIA commercial kit  - radioimmunoassay | Binding assay | 100 (161) | 8.00 a.m. - 10.00 a.m. | 20.5 (5.9) | 24.9 (10.1) | 507.4 (138.1) |
| Undén et al, 2005 [36] | RIA commercial kit  - radioimmunoassay | AutoDelfia  - fluoroimmunoassay | 100 (137) | 8.00 a.m. - 12.00 a.m. | 19.1 (6.8) | 44.5 (21.9) | 349.1 (139.7) |
| Tong et al, 2005 [37] | Immulite  - chemiluminescent immunoassay | Immulite  - chemiluminescent immunoassay | 100 (295) | 8.00 a.m. - 10.00 a.m. | 18.1 (5.6) | 29.2 (13.2) | 413.8 (112.7) |
| Laaksonen et al, 2004 [15] | AutoDelfia  - fluoroimmunoassay | AutoDelfia  - fluoroimmunoassay | 100 (2028) | 8.00 a.m. - 10.00 a.m. | 20.4 (7.2) | 38.7 (16.6) | 399.7 (113.0) |
| Ukkola et al, 2001 [38] | In house-RIA with hexane ethyl acetate extraction  - radioimmunoassay | Diagnostics Systems Laboratories  - immunoradiometric assay | 100 (321) | NA | 15.1 (5.9) | 38.4 (16.5) | 292.0 (115.8) |
| Hautanen et al, 2000 ^35^ | RIA commercial kit  - radioimmunoassay | Delfia  - immunoradiometric assay | 100 (96) | 7.30 a.m. | 17.3 (5.3) | 38.1 (16.0) | 335.3 (97.7) |

Abbreviations: RIA; radioimmunoassay; SHBG = Sex hormone-binding globulin; SD = standard deviation; NA = not available.
